# Supplementary material for: Altered subgenomic RNA abundance provides unique insight into SARS-CoV-2 B.1.1.7/Alpha variant infections
Source: Commun Biol. 2022 Jul 5;5:666. doi: 10.1038/s42003-022-03565-9 (PMC9255483; doi:10.1038/s42003-022-03565-9)
Supplement: Supplementary file 12 — Reporting Summary [file 42003_2022_3565_MOESM12_ESM.pdf]

nature portfolio

Corresponding author(s): Thushan de Silva

Last updated by author(s): Jan 5, 2022

# Reporting Summary

Nature Portfolio wishes to improve the reproducibility of the work that we publish. This form provides structure for consistency and transparency in reporting. For further information on Nature Portfolio policies, see our Editorial Policies and the Editorial Policy Checklist.

## Statistics

For all statistical analyses, confirm that the following items are present in the figure legend, table legend, main text, or Methods section.

| n/a | Confirmed | |
|---|---|---|
| ☐ | ☒ | The exact sample size ($n$) for each experimental group/condition, given as a discrete number and unit of measurement |
| ☐ | ☒ | A statement on whether measurements were taken from distinct samples or whether the same sample was measured repeatedly |
| ☐ | ☒ | The statistical test(s) used AND whether they are one- or two-sided<br>*Only common tests should be described solely by name; describe more complex techniques in the Methods section.* |
| ☐ | ☒ | A description of all covariates tested |
| ☐ | ☒ | A description of any assumptions or corrections, such as tests of normality and adjustment for multiple comparisons |
| ☐ | ☒ | A full description of the statistical parameters including central tendency (e.g. means) or other basic estimates (e.g. regression coefficient) AND variation (e.g. standard deviation) or associated estimates of uncertainty (e.g. confidence intervals) |
| ☐ | ☒ | For null hypothesis testing, the test statistic (e.g. $F$, $t$, $r$) with confidence intervals, effect sizes, degrees of freedom and $P$ value noted<br>*Give P values as exact values whenever suitable.* |
| ☒ | ☐ | For Bayesian analysis, information on the choice of priors and Markov chain Monte Carlo settings |
| ☒ | ☐ | For hierarchical and complex designs, identification of the appropriate level for tests and full reporting of outcomes |
| ☒ | ☐ | Estimates of effect sizes (e.g. Cohen's $d$, Pearson's $r$), indicating how they were calculated |

*Our web collection on statistics for biologists contains articles on many of the points above.*

## Software and code

Policy information about availability of computer code

| | |
|---|---|
| Data collection | Periscope is available at https://github.com/sheffield-bioinformatics-core/periscope and version 0.0.8a used in this publication is available at doi://10.5281/zenodo.5717129 the code used to generate the figures contained within this manuscript can be found as a supplementary data, at https://github.com/sheffield-bioinformatics-core/periscope-variants-publication or also available at doi://10.5281/zenodo.5717129 |
| Data analysis | As above |

For manuscripts utilizing custom algorithms or software that are central to the research but not yet described in published literature, software must be made available to editors and reviewers. We strongly encourage code deposition in a community repository (e.g. GitHub). See the Nature Portfolio guidelines for submitting code & software for further information.

## Data

Policy information about availability of data

All manuscripts must include a data availability statement. This statement should provide the following information, where applicable:

- Accession codes, unique identifiers, or web links for publicly available datasets
- A description of any restrictions on data availability
- For clinical datasets or third party data, please ensure that the statement adheres to our policy

All SARS-CoV-2 consensus sequences that are of high enough quality are available on GISAID and ENA and from https://www.cogconsortium.uk/data/. All sgRNA abundance data is provided as supplementary data and at https://github.com/sheffield-bioinformatics-core/periscope-variants-publication. All raw sequencing data are available on ENA under the accession PRJEB48895.

The mass spectrometry proteomics data has been deposited to the ProteomeXchange Consortium via the PRIDE partner repository with the dataset identifier PXD029954.

# Field-specific reporting

Please select the one below that is the best fit for your research. If you are not sure, read the appropriate sections before making your selection.

- [x] Life sciences
- [ ] Behavioural & social sciences
- [ ] Ecological, evolutionary & environmental sciences

For a reference copy of the document with all sections, see nature.com/documents/nr-reporting-summary-flat.pdf

# Life sciences study design

All studies must disclose on these points even when the disclosure is negative.

| | |
|---|---|
| Sample size | Sample size was determined by the total number of sequenced samples available. |
| Data exclusions | The only exclusions made was on the basis of sequence data quality, which is clearly described in the manuscript |
| Replication | NA |
| Randomization | NA |
| Blinding | NA |

# Reporting for specific materials, systems and methods

We require information from authors about some types of materials, experimental systems and methods used in many studies. Here, indicate whether each material, system or method listed is relevant to your study. If you are not sure if a list item applies to your research, read the appropriate section before selecting a response.

## Materials & experimental systems

| n/a | Involved in the study | |
|---|---|---|
| x | | Antibodies |
| x | | Eukaryotic cell lines |
| x | | Palaeontology and archaeology |
| x | | Animals and other organisms |
| | x | Human research participants |
| x | | Clinical data |
| x | | Dual use research of concern |

## Methods

| n/a | Involved in the study | |
|---|---|---|
| x | | ChIP-seq |
| x | | Flow cytometry |
| x | | MRI-based neuroimaging |

## Human research participants

Policy information about studies involving human research participants

| | |
|---|---|
| Population characteristics | Individuals presenting with active COVID-19 disease were sampled for SARS CoV-2 sequencing at Sheffield Teaching Hospitals NHS Foundation Trust, UK using samples collected for routine clinical diagnostic use. |
| Recruitment | No individual participant recruitment was performed. Sequences were generated from individuals presenting with clinical SARS-CoV-2 infections |
| Ethics oversight | This work was performed under approval by the Public Health England Research Ethics and Governance Group for the COVID-19 Genomics UK consortium (R&D NR0195). Approval was provided to undertake viral sequencing on residual clinical diagnostic samples and analysis on anonymised data without individual patients consent. |

Note that full information on the approval of the study protocol must also be provided in the manuscript.
