# Supplementary material for: Altered subgenomic RNA abundance provides unique insight into SARS-CoV-2 B.1.1.7/Alpha variant infections
Source: Commun Biol. 2022 Jul 5;5:666. doi: 10.1038/s42003-022-03565-9 (PMC9255483; doi:10.1038/s42003-022-03565-9)
Supplement: Supplementary file 13 — Description of Additional Supplementary Files [file 42003_2022_3565_MOESM13_ESM.pdf]

File Name: Supplementary Data 1
Description: Pillar 1 genome metadata

File Name: Supplementary Data 2
Description: Pillar 2 genome metadata

File Name: Supplementary Data 3
Description: Periscope canonical sgRNA counts for Pillar 1 samples

File Name: Supplementary Data 4
Description: Periscope canonical sgRNA counts for Pillar 2 samples

File Name: Supplementary Data 5
Description: LC-MS/MS protein abundance data

File Name: Supplementary Data 6
Description: Pillar 1 sample days since symptom onset

File Name: Supplementary Data 7
Description: Periscope noncanonical sgRNA counts for Pillar 1 samples. Note: some samples do not have any predicted noncanonical sgRNA and so will not appear in this file.

File Name: Supplementary Data 8
Description: ARTIC Network SARS-CoV-2 V3 primer sequences

File Name: Supplementary Data 9
Description: R code to recreate figures and analyses
